# Weakness after Urination

**Published:** 1890-11

**Authors:** Robert Farley

**Affiliations:** Phœnixville, Pa.


### WEAKNESS AFTER URINATION.

EDITOR HOMŒOPATHIC PHYSICIAN:—After prescribing several seemingly indicated remedies for a male patient, a few months ago, with no curative result, I learned—or rather he expressed himself differently in trying to convey the idea or sensation he had previously expressed in other terms—that he suffered after urination with a feeling of weakness, relaxation, very distressing in character, in the sexual organs; said he must have seminal emissions each time he urinated, as he felt just as he used to after masturbating. This condition I found to be met by Berb-vulg., which I gave him in the CM potency, and improvement began with the next act of urination. My patient was so distressed by the condition that he was becoming melancholy. The case as I took it for this prescription very strongly suggested Staph., but this peculiar condition of distress and weakness after urination made me decide in favor of Berb-v., with a very happy result and a verification of the recorded condition.

PHŒNIXVILLE, PA. ROBT. FARLEY, M. D.

